# ﻿*Lappulamonocarpa*, a new synonym of *Lappulatenuis* (Boraginaceae)

**DOI:** 10.3897/phytokeys.259.157867

**Published:** 2025-07-04

**Authors:** Dan-Hui Liu

**Affiliations:** 1 State Key Laboratory of Ecological Safety and Sustainable Development in Arid Lands, Xinjiang Institute of Ecology and Geography, Chinese Academy of Sciences, Urumqi, 830011, Xinjiang, China Xinjiang Institute of Ecology and Geography, Chinese Academy of Sciences Urumqi China; 2 Xinjiang Key Lab of Conservation and Utilization of Plant Gene Resources, Xinjiang Institute of Ecology and Geography, Chinese Academy of Sciences, Urumqi 830011, Xinjiang, China Xinjiang Institute of Ecology and Geography, Chinese Academy of Sciences Urumqi China; 3 Xinjiang Key Laboratory of Biodiversity Conservation and Application in Arid Lands, Xinjiang Institute of Ecology and Geography, Chinese Academy of Sciences, Urumqi, 830011, Xinjiang, China Xinjiang Institute of Ecology and Geography, Chinese Academy of Sciences Urumqi China

**Keywords:** Boraginaceae, *Lappula*, morphology, new synonym

## Abstract

*Lappulamonocarpa* is currently the sole described species within the genus *Lappula* characterized by the development of a single nutlet per fruit. However, since its initial publication, no additional specimens of *L.monocarpa* have been collected. Critical examination of type specimens, combined with field surveys and newly collected material, reveals that the diagnostic trait of *L.monocarpa*—the presence of a single nutlet per fruit—is not consistently observed and likely represents an aberrant specimen. Moreover, all other morphological characteristics of *L.monocarpa*, including indumentum, corolla, and nutlet features, align fully with those of *L.tenuis*. Based on this evidence, *L.monocarpa* is herein reduced to a synonym of *L.tenuis*.

## ﻿Introduction

*Lappula* Moench is one of the largest genera within the tribe Rochelieae of the family Boraginaceae (Chacón et al. 2016; Vasile et al. 2025). It comprises approximately 50 to 80 species distributed across Eurasia, North Africa, North and South America, and Australia, with a center of diversity located in Central Asia (Wang 1981; Ovczinnikova 2005, 2021; Weigend et al. 2016). Members of *Lappula* are characterized by blue or white corollas with five throat appendages, a subulate gynobase, and heteromorphic or homomorphic nutlets bearing marginal glochids or wings (Popov 1953; Riedl 1967; Wang 1981; Ovczinnikova 2005). In China, 36 species of *Lappula* have been recorded (Zhu et al. 1995), of which at least nine are endemic.

*Lappulamonocarpa* C.J.Wang is a Chinese endemic species, restricted to Xinjiang Province (Wang 1981). It was originally described based on a specimen collected from Heishantou (Black Mountain), Jimunai County, Xinjiang. The species is distinguished by the production of a single, horizontally oriented nutlet per fruit, with a disc margin bearing a single row of glochids. In the protologue, Wang (1981) emphasized that these morphological traits are highly distinctive within *Lappula*, particularly the presence of a solitary nutlet, which clearly separates *L.monocarpa* from all other taxa of *Lappula*. Concurrent with the species description, Wang (1981) also established a new monotypic subsection, Lappulasubsect.Monocarpae C.J.Wang, to accommodate it. However, since its original description, no additional specimens of *L.monocarpa* have been collected beyond the type specimen.

The nutlets of *L.monocarpa* bear a single row of marginal glochids with non-connate bases. Based on these traits, Ovczinnikova (2005) assigned *L.monocarpa* to ser. Strictae, a classification that contradicts Wang’s (1981) earlier taxonomic treatment. Subsequently, Ovczinnikova (2009) proposed that *L.monocarpa* may represent only a variant of *L.stricta*, distinguished solely by its production of a single nutlet per fruit. Nutlet characteristics are extensively utilized for infrageneric classification and species delineation within *Lappula*. These characteristics include the presence of glochids or wings, the number of glochid rows, the length of the glochids, the width of the wings, and the shape of the dorsal disk (Gürke 1894; Popov 1953; Riedl 1967; Wang 1981; Ovczinnikova 2005, 2009, 2021). However, the number of nutlets per fruit has not been employed as a diagnostic character for species delineation, as all currently described species of *Lappula* typically develop four nutlets per fruit, with the exception of *L.monocarpa* (Popov 1953; Wang 1981; Ovczinnikova 2005, 2021, 2023; Liu et al. 2024).

**Figure 1.**
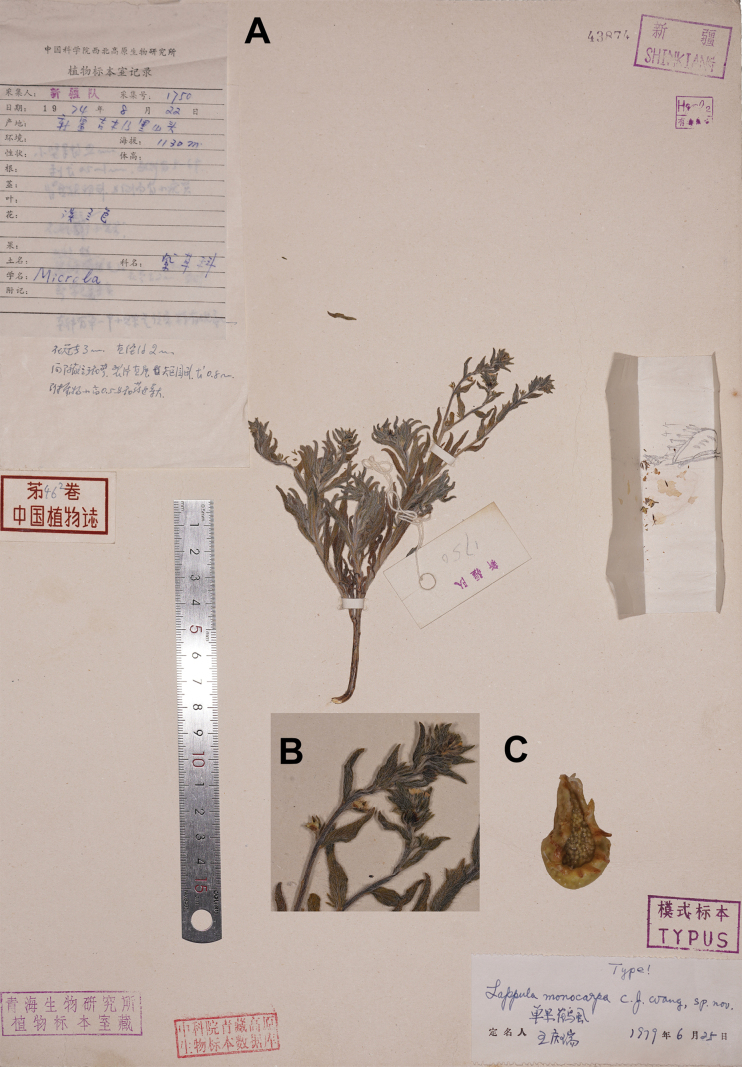
Type specimen of *Lappulamonocarpa* C.J.Wang. **A.** Holotype in the herbarium of the Northwest Institute of Plateau Biology, Chinese Academy of Sciences (HNWP); **B, C.** Details of the holotype; **B.** Development of a single nutlet per fruit, with the nutlet horizontally oriented; **C.** Nutlet morphology.

Further examination of the type specimen of *L.monocarpa* revealed that it bears only a few developing fruits, each containing a single nutlet. This nutlet is ovoid and features a single row of glochids (Fig. 1). Apart from the difference in nutlet number, all other nutlet characteristics align fully with those of *L.tenuis* (Ledeb.) Gürke. These observations suggest that *L.monocarpa* and *L.tenuis* represent the same taxonomic entity. To test this hypothesis, field surveys were conducted at the type locality of *L.monocarpa*, and additional specimens were collected. Morphological comparisons among *L.monocarpa*, *L.tenuis*, and *L.stricta* (Ledeb.) Gürke were then carried out to clarify the taxonomic status of *L.monocarpa*.

## ﻿Material and methods

Herbarium specimens of *Lappula* from ALTB, BNU, HNWP, KUZ, LE, and NSK were thoroughly examined, including the type specimens of *L.monocarpa* and *L.tenuis*. A field survey was conducted at the type locality of *L.monocarpa* in Heishantou (Black Mountain), Jimunai County, Xinjiang Province, China, where additional specimens were collected and deposited at BNU. Detailed morphological features, including stem indumentum, calyx, corolla, nutlets, and gynobase, were documented *in situ* using a Sony Alpha 7 camera. Comparative morphological analyses of *L.monocarpa*, *L.stricta*, and *L.tenuis* were performed to assess their taxonomic boundaries.

## ﻿Results

**Figure 2.**
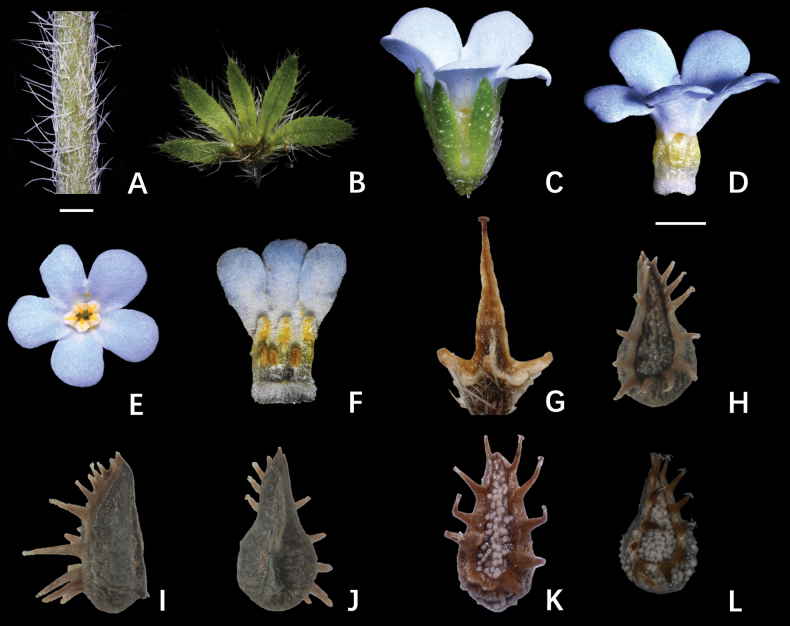
Morphological features of *Lappulamonocarpa* C.J.Wang (**A–J**) and nutlet morphology of *L.stricta* (Ledeb.) Gürke (**K**) and *L.tenuis* (Ledeb.) Gürke (**L**). **A.** Characteristics of stem trichomes, showing appressed and spreading hairs; **B.** Calyx; **C, D, E, F.** Flower morphology; **C, D.** Lateral view of the flower; **E.** Top view of the flower; **F.** Expanded flower morphology, showing the corolla throat appendages and stamens; **G.** Gynobase morphology; **H, I, J.** Nutlet morphology; **H.** Abaxial view of the nutlet; **I.** Lateral view of the nutlet; **J.** Adaxial view of the nutlet; **K.** Nutlet morphology of *L.stricta*; **L.** Nutlet morphology of *L.tenuis*. Scale bars: 1 mm.

During field investigations, no *Lappula* specimens matching the original description of producing a single nutlet per fruit were encountered. All collected individuals consistently developed four nutlets per fruit. Aside from this numerical discrepancy, their morphological features were indistinguishable from those described for *L.monocarpa*. Detailed morphological comparisons among *L.monocarpa*, *L.stricta*, and *L.tenuis* revealed congruent characteristics in stem indumentum, bract morphology, calyx, and corolla (Fig. 2, Table 1). The primary diagnostic differences were confined to nutlet morphology: both *L.monocarpa* and *L.tenuis* exhibit ovate dorsal disks (length-to-width ratios of 2.5–3), with the greatest width occurring below the midpoint. These nutlets lack a disk keel and possess non-thickened margins. In contrast, nutlets of *L.stricta* are lanceolate (length-to-width ratio ~4), may or may not display a disk keel, and typically have thickened margins.

**Table 1.** Morphological comparisons of *Lappulamonocarpa*, *L.stricta*, and *L.tenuis*.

Characters		*L.monocarpa*	*L.stricta*	*L.tenuis*
Life form		annual	annual	annual
Indumentum		spreading and appressed hair	spreading and appressed hair	spreading and appressed hair
Bracts		leaf-like at the base of the inflorescence, gradually reduced toward the apex	leaf-like at the base of the inflorescence, gradually reduced toward the apex	leaf-like at the base of the inflorescence, gradually reduced toward the apex
Calyx	lobes	linear	linear	linear
	length	exceeding the fruit	exceeding the fruit	exceeding the fruit
Corolla	length	ca. 3 mm	ca. 3 mm	ca. 3 mm
Nutlet	glochids	single row	single row	single row
	disk shape	ovate	lanceolate	ovate
	disk keel	without	with or without	without
	margin	not thickened	thickened	not thickened
Style		slightly surpassing nutlet	slightly surpassing nutlet	slightly surpassing nutlet

## ﻿Discussion

Ovczinnikova (2005) circumscribed Lappulasect.Lappula to include five series: ser. Lappula, ser. Redowskianae Ovczinnikova, ser. Anisacanthae Ovczinnikova, ser. Strictae M.Pop. ex Ovczinnikova, and ser. Patulae Ovczinnikova (Table 2). Recently, Liu et al. (2025) reconstructed the phylogenetic relationships of Asian *Lappula* using hundreds of single-copy nuclear genes and complete chloroplast genomes. Their results support the monophyly of sect. Lappula as defined by Ovczinnikova (2005). However, the placement of certain taxa conflicts with this classification. Notably, *L.monocarpa* clustered with *L.consanguinea* and *L.anocarpa*, indicating a closer affinity with ser. Lappula. Furthermore, *L.monocarpa* and *L.stricta* occupy distinct, strongly supported subclades within sect. Lappula (Clade IV; Liu et al. 2025). This phylogenetic topology supports neither the inclusion of *L.monocarpa* in ser. Strictae (Ovczinnikova 2005), nor the hypothesis that *L.monocarpa* represents a variant of *L.stricta* (Ovczinnikova 2009).

**Table 2.** The subdivision of Lappulasect.Lappula by Ovczinnikova (2005), including the species composition of each series.

	Sect. Lappula				
	Ser. Lappula	Ser. Redowskianae	Ser. Anisacanthae	Ser. Strictae	Ser. Patulae
Taxa	*L.brachycentroides*	*L.occidentalis*	*L.anisacantha*	*L.caespitosa*	*L.patula*
	*L.consanguinea*	*L.redowskii*	*L.lenensis*	*L.coronifera*	
	*L.heteracantha*		*L.shanhsiensis*	*L.cristata*	
	*L.intermedia*			*L.fruticulosa*	
	*L.squarrosa*			*L.karelinii*	
	*L.tenuis*			*L.marginata*	
	*L.tuvinica*			*L.monocarpa*	
				*L.physacantha*	
				*L.stricta*	
				*L.zaissanica*	

Ovczinnikova (2005) defined ser. Strictae as comprising taxa with nutlets bearing a single row of glochids, typically with broadened bases or, occasionally, forming marginal wings. In contrast, ser. Lappula includes nutlets with one to several rows of glochids, the bases of which are neither broadened nor winged. Ovczinnikova assigned *L.monocarpa* to ser. Strictae and *L.tenuis* to ser. Lappula. However, both the type specimen of *L.monocarpa* and newly collected material from its type locality reveal that the glochids have neither broadened nor winged bases, and no winged nutlets were observed in the population. These morphological traits are inconsistent with the defining characteristics of ser. Strictae.

Detailed morphological comparisons between the Asian *Lappula* taxa *L.stricta* (ser. Strictae) and *L.tenuis* (ser. Lappula) reveal that the primary distinguishing feature between these two series lies in the shape of the nutlet disk, rather than in the number of glochid rows. The “disk” refers to the flattened area on the dorsal side of the nutlet, the margin of which typically bears glochids or wings (Hilger 2014). Nutlets of ser. Strictae are lanceolate and may or may not possess a keel, whereas those of ser. Lappula are ovate and consistently keel-less. The nutlet disk of *L.monocarpa* is ovate and lacks a keel, supporting its placement within ser. Lappula on morphological grounds, in agreement with the phylogenetic results of Liu et al. (2025). Additionally, some North American *Lappula* taxa, such as *L.desertorum* Greene and *L.heterosperma* Greene, exhibit a ridge on the dorsal face of the nutlet or lines of tubercles along the midline of the disk (Rolfsmeier 2013). Liu et al. (2025) suggested that these North American taxa may be closely related to Clade IV on the Eurasian continent. The species examined in the present study, *L.monocarpa* and *L.stricta*, also belong to Clade IV. While sect. Lappula, as defined by Ovczinnikova (2005), includes only one native North American species, *L.occidentalis* (S. Watson) Greene, the phylogenetic placement of other North American *Lappula* taxa remains uncertain and requires further investigation using both molecular and morphological data.

As defined by Ovczinnikova (2005), ser. Lappula comprises seven species, among which only *L.tenuis* and *L.brachycentroides* Popov produce nutlets with a single row of spines. Popov (1951) noted that *L.brachycentroides* is closely related to *Echinospermumtenue* Ledeb. (now accepted as *L.tenuis*), differing primarily by the presence of short or absent glochids on the nutlets. The nutlets of *L.monocarpa* bear a single row of glochids with non-broadened bases and exhibit an ovate, non-lanceolate dorsal disk. Taken together, the molecular phylogenetic findings of Liu et al. (2025) and the morphological evidence presented here support the placement of *L.monocarpa* within ser. Lappula. This classification supports the reduction of *L.monocarpa* to a taxonomic synonym of *L.tenuis*.

### ﻿Taxonomic treatment

#### ﻿ Lappula tenuis

Taxon classificationPlantaeBoraginalesBoraginaceae

(Ledeb.) Gürke, Nat. Pflanzenfam. 4(3a): 107, 1894

1C10493C-9ACA-5EEA-B92C-8ED01ECE6EAB

≡ Echinospermumtenue Ledeb, Fl. Altaic. 1: 201, 1829.
≡ Hackeliatenuis (Ledeb.) Opiz, Oekon.-Techn. Fl. Böhm. 2(2): 147, 1839.
≡ Myosotistenuis (Ledeb.) Mörch, Cat. Hort. Hafn.: 64, 1839.
≡ Echinospermumredowskiivar.tenue (Ledeb.) Regel, Bull. Soc. Imp. Naturalistes Moscou 41(I): 84, 1868.
≡ Cynoglossospermumtenue (Ledeb.) Kuntze, Revis. Gen. Pl. 2: 437, 1891.
= Lappulamonocarpa C.J.Wang, Bull. Bot. Res., Harbin 1(4): 98, 1981. syn. nov. Type. China, Xinjiang province, Jimunai County, 1130 m a.s.l., 22 August 1974, Xinjiang Exped. 1750 (Holotype: HNWP!).

##### Type.

Russia • Altai, Ad fluv. Tscharysch, 1826, Ledebour, Smejow and Politow s. n. (Lecotype by Ovczinnikova (2018), ***Lectotype***: LE01043915!).

##### Distribution and habitat.

*Lappulatenuis* is distributed in China, Kazakhstan, and Russia (Wang 1981, Ovczinnikova 2009, Chepinoga et al. 2024). This species inhabits grasslands and steppes at altitudes of approximately 1,000 meters above sea level.

##### Phenology.

Flowering and fruiting from June to August.

##### Taxonomic notes.

Fruits of *Lappula* typically develop four nutlets; however, an extensive review of herbarium specimens revealed that some individuals, for example, *L.caespitosa* C.J.Wang (BNU0033484) and *L.microcarpa* (Ledeb.) Gürke (BNU0030476, BNU0030622, E00843758), sometimes produce only a single developed nutlet. Due to the infrequent and irregular occurrence of such aberrations, nutlet number is considered an unreliable diagnostic character for species delimitation within the genus. During field surveys, *L.tenuis* was occasionally found growing sympatrically with *L.consanguinea* (Fisch. & C.A.Mey.) Gürke and *L.anocarpa* C.J.Wang. Although these three species share similar vegetative morphology and ovoid nutlet shape, they can be reliably distinguished by the arrangement of glochids: *L.tenuis* has nutlets with a single row of marginal glochids, whereas *L.consanguinea* and *L.anocarpa* bear two or three rows.

Ovczinnikova (2005) circumscribed ser. Lappula to include only two species with single-rowed marginal glochids: *L.tenuis* and *L.brachycentroides*. Popov (1951) recognized the morphological similarity between these taxa but distinguished *L.brachycentroides* by the presence of short or absent glochids along the nutlet margins. However, my examination of *L.brachycentroides* specimens revealed that nutlets on the same individual can exhibit either smooth margins or distinct glochids, with the latter morphology being consistent with that of *L.tenuis*. Fruit heteromorphism is well documented in the genus *Lappula*, including variation in glochid presence (glochids or wings, long-glochids, short-glochids, or glochidless) and wing morphology (broad- or narrow-winged). These observations suggest that *L.brachycentroides* may simply represent a fruit-dimorphic form of *L.tenuis*. However, as no newly collected material of *L.brachycentroides* was obtained in this study, the taxonomic status of this species requires further investigation.

##### Additional specimens examined.

**China. Xinjiang**: • Altai, *Xinjiang Exped. 10669* (PE01361016); • Jimunai County, *D.H. Liu BNU2019XJ214* (BNU); • Qinghe County, *D.H. Liu BNU2019XJ182* (BNU). **Kazakhstan.** • Akmola region, *Lashchinsky N.N. s.n.* (NSK0009103); • East Kazakhstan, *Kotukhov A.Yu. s.n.* (NSK0008186); • Zharminsky district, *Korolyuk A.Yu. 132* (NSK0005792); • Kokpekti district, *Popov N.V. 1* (NSK0005797); **Russia.** • Altai region, *Usyk N.A. s.n.* (ALTB1100059605, ALTB1100060899); • Kemerovo region, *Strelnikova T.O.* and *Manakov Y.A. KEM18465* (KUZ026052); • Kosh-Agachsky district, *Korolyuk A.Yu. 121* (NSK0005798); • Ongudaysky district, *Maslova O.M.* and *Khrustaleva I.A. s.n.* (NSK0005819); • Orenburg region, *Lashchinsky N.N. L15-174* (NSK0009078).

## Supplementary Material

XML Treatment for Lappula tenuis

## ﻿Additional information

### Use of AI

No use of AI was reported.

### Author ORCIDs

Dan-Hui Liu https://orcid.org/0000-0002-0195-1436

### Data availability

All of the data that support the findings of this study are available in the main text.
